# Pain in Tourette Syndrome-Children’s and Parents’ Perspectives

**DOI:** 10.3390/jcm11020460

**Published:** 2022-01-17

**Authors:** Agnieszka Małek

**Affiliations:** Department of Physical Education and Social Sciences, Gdansk University of Physical Education and Sport, 80-336 Gdansk, Poland; agnieszka.malek@awf.gda.pl; Tel.: +48-88-390-7387

**Keywords:** Tourette Syndrome (TS), tic, chronic disorder, pain, pain intensity, pain coping strategies

## Abstract

Tourette Syndrome (TS) is a neurodevelopmental condition characterized by the presence of tics and associated behavioral problems. Yale Global Tic Severity Scale (YGTSS), The PedsQL Pediatric Pain Questionnaire, and Pediatric Pain Coping Inventory were used to assess the severity of tics, the severity of the pain, the location of the pain and pain coping strategies both from children’s and parents’ perspectives. Sixty percent of children demonstrated pain (past or present); the pain was reported by 72% of parents raising children with TS. The pain most commonly was cervical, throat, shoulder, ocular, and joint pain; most children declared pain located in more than one part of the body. Consistency between the declarations of children and their parents in coping with pain was observed. Pain should be recognized as a common comorbid aspect of tic disorders in childhood and therapeutic treatment must include the reduction of pain caused by tics.

## 1. Introduction

Tourette Syndrome (TS) is a neurodevelopmental disorder characterized by co-occurring motor tics together with a minimum of one vocal tic, occurring several times a day or periodically for a period of at least one year after the first episode of the disorder [1]. It is estimated that the condition affects about 1% of the population [2]. According to data from screening tests conducted in 2000, TS may affect from 0.43% to 3.8% of Polish students [3]. Until now, the exact cause of TS is not fully understood—the hypothesis about the inherited dysfunction of the basal ganglia and the cortico-striatal-thalamic-loop seems to be the most probable explanation [4,5]. The syndrome is also known to coexist with various psychiatric comorbidities, such as attention-deficit/hyperactivity disorder (ADHD), obsessive-compulsive disorder (OCD) and its variant, obsessive-compulsive symptoms (OCS), depression, anxiety disorders, impulse control disorders (ICD), or learning difficulties [6,7,8].

Tics, both motor and vocal, may be associated with pain. In adults suffering from TS, this association may be as high as 47.5% [9]. The pain was also recognized as a common comorbid aspect of tic disorders in childhood: 15–20% [10] of children with tics/TS demonstrated pain. The following quote: “The pain most commonly was ocular, cervical, followed by shoulder, back, abdominal, and joint pain. Carpal tunnel pain also was described (…). While most pain originated as musculoskeletal in nature, neuropathic pain also was reported. Pain generally was in association with the voluntary movement or in an attempt to suppress their tics” [10] concludes Lavenstein, Miyares, and Dodge’s findings regarding pain in TS. It was also found that the prevalence of headache in patients with TS could be higher than in the general population, with the most reliable data being available for migraine [11]. In extreme cases, pain may be caused by soft tissue damage (muscles, ligaments, or tendons) due to forceful tics [12]. The pain/Discomfort dimension was the second largest factor of decreased quality of life [9]. Riley and Lang [13] presented the following classification of pain related to tics and/or compulsions in patients with TS and OCD:

As reported patients have experienced more than one type of pain as categorized in Table 1; the pain was often the symptom of greatest concern. Painful tics are a common cause of the decision to initiate pharmacologic intervention [10,14]. Evidence-based knowledge on this aspect of TS would enable clinicians to improve diagnostic, therapeutic, and parental strategies for child patients.

The aims of the study were: to assess pain in children with TS, as reported by the children, and their parents; to compare agreement between child and parent concerning pain intensity and localization; to find children’s pain coping strategies, and to compare compatibility of children’s and their parents’ perceptions of needs and ways of supporting pain caused by tics.

## 2. Materials and Methods

The study was approved by the Bioethics Committee of the Regional Medical Chamber of Gdańsk, Poland (KB-47/21).

The following data were collected during August-September 2021. All participants (both children and parents) were familiarized with the purpose of the study and agreed to participate in the project. The inclusion criteria were as follows: children—diagnosis of TS made by a neurologist according to the DSM-5 criteria, age 5–18; adults—being a parent of a child participating in the study.

**Figure 1:**
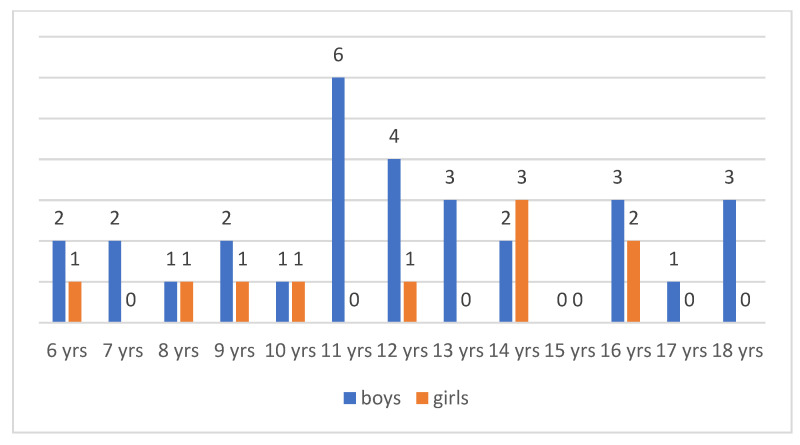
Age of children with TS.

97 participants took part in the project—40 children (31 boys and 9 girls) aged 6–18 years (*M* = 12, *SD* = 3.43) (the number of respondents in each age group is shown in Figure 1) and their parents (57 individuals: 35 mothers and 22 fathers). 

**Figure 2:**
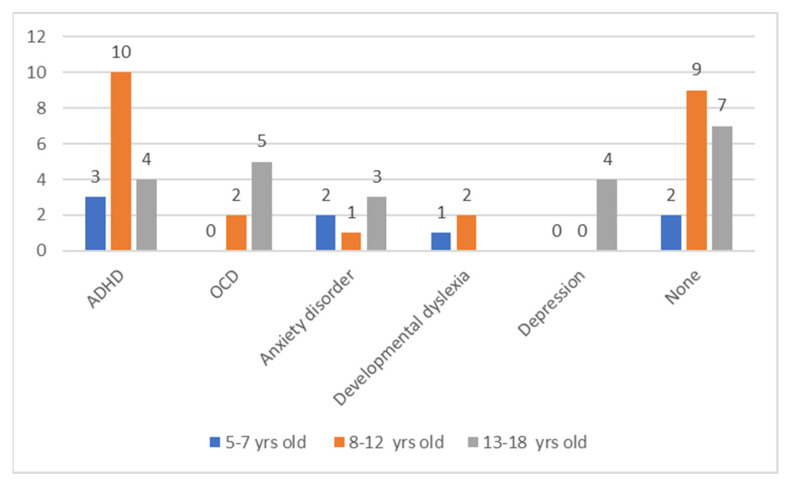
Comorbid conditions. ADHD—Attention-Deficit/Hyperactivity Disorder; OCD—Obsessive-Compulsive Disorder.

An interview was conducted, during which the subjects declared the number and type of co-morbidities. Several children reported the occurrence of more than one condition. The frequency of the coexistence of individual disorders is presented in the figure below (Figure 2).

Tics were assessed with The Polish adaptation of the Yale Global Tic Severity Scale (YGTSS) [15,16]. YGTSS consists of two parts: the first one contains a list of simple and complex motor and vocal tics present during the last week. The second part is used to assess the number, frequency, severity, and complexity of tics, as well as the general impairment of everyday functioning; the second part of the questionnaire contains separate subscales for motor and vocal tics. Responses are scored on a scale from 0 to 5; in total, 100 points can be obtained. While interpreting the YGTSS, we should assume that high scores obtained by the patient in the examination suggest a greater severity of the tic disorder and a more significant impairment of daily life.

The PedsQL Pediatric Pain Questionnaire [17] was used to measure the intensity and the localization of pain. Forms are available for three age groups: age 5–7, age 8 to 12, and age 13 to 18. The PedsQL is a visual analogue scale (VAS) and is composed of 3 items: Present Pain, Worst Pain, and localization of pain. Pain intensity dimensions are scored from 0 (Not hurting/No discomfort/No pain) to 10 (Hurting a whole lot/Very uncomfortable/Severe pain). The third item refers to the localization of pain and is not scored. Both children’s and parents’ versions were used.

The study used the Pediatric Pain Coping Inventory—a specific module of PedsQL [18] to find what children do when they are in pain (pain coping strategies). Existing versions of the questionnaire: Child (5–12 years of age), Adolescent (13–18 years of age), and Parents were used. 

Information on the drugs used (currently or in the past) to reduce tics and participation in psychological therapy were also taken. Fifteen children received medicines at the time of the study, other’s parents declared that their children never used pharmacological therapy. Among children participating in the study, thirty-one participated in the individual cognitive-behavioral therapy.

## 3. Results

Statistical analysis: all analyses were performed using IBM SPSS Statistics software version 27.0 (IBM SPSS Statistics for Windows, Armonk, NY, USA). Non-parametric tests were used because all the variables were not normally distributed.

### 3.1. Pain Severity

In an analysis of 40 children with TS 24 of them (60%) demonstrated pain (past or present). Pain was reported by 41 (72) % of parents raising children with TS. The correlations between the scores of pain intensity and tic severity are reported in Table 2.

Within each group of respondents, significant correlations were found between present pain and tic severity, and worst pain and tic severity (Spearman’s rho). 

To assess whether there is a difference for children and their parents in pain intensity assessment, both present and past, The Mann-Whitney U test was used. There was no statistically significant difference between groups (Table 3, Table 4 and Table 5).

### 3.2. Location of Pain

The pain most commonly was cervical, throat, shoulder, ocular, and joint pain. Most children declared pain located in more than one part of the body. Parents’ responses to the localization of pain were consistent with the children’s declarations.

### 3.3. Pain Descriptors

Pain descriptor was also asked. Children and parents tended to use many of the same words to describe the children’s pain. The most frequently selected pain descriptors are reported in Table 6. 

### 3.4. Pain Coping Strategies

To find children’s pain coping strategies, we asked both children and parents to answer the questions about what they usually do when feeling the pain. The responses were then assigned to the following categories: seek for social support and action, cognitive self-instruction, distraction, and problem-solving [18] (due to Pediatric Pain Coping Inventory). Cognitive self-instruction strategy is the most often presented by younger children (5–12 years) with TS. They also use other active pain coping strategies: asking for help, and distraction. Parents were also asked to describe their child’s pain coping strategies to compare the parental perception of the child’s needs when in pain. The most frequently selected by younger children and their parents’ pain coping strategies are reported in Table 7.

Both adolescents and their parents were also asked to describe pain coping strategies. Adolescents (13–18 years) preferred both passive (resting) and active pain coping strategies (distraction or pain transformation). They prefer isolation during pain when younger children declared the need to involve parents when struggling with pain. The pain coping strategies most frequently selected by adolescents are reported in Table 8.

In general, an agreement between mostly pointed behaviors during pain can be found in both age groups. Parents usually know about their child’s needs when in pain, especially younger ones.

## 4. Discussion

TS is a chronic disorder that affects patients’ everyday life. Pain is rarely discussed in the context of tic disorders, but it was found in this study as a common phenomenon associated with TS in childhood. The results showed a significantly higher prevalence of pain than earlier described in the literature [10]. Based on the results obtained, it is concluded that the severity of tics is related to the intensity of pain. It could be explained by individual disease characteristics: the higher the number, frequency, severity, and complexity of tics, the higher the risk of pain. The pain experienced by the child may also influence the declared impairment of everyday functioning, which is in accordance with data collected from adults suffering from tics [9]. It is well known that pain intensity affects the psychological well-being of patients with chronic pain [19].

Our results concerning the location of pain were all in compliance with those earlier described in the literature [10,12]. The most common was the exertional type of pain, including muscular pain due to excessive contraction and joint pain. All children with pain declared that more than one part of the body was affected.

As claimed in the previous studies [20,21] we found that children and adolescents declared lower intensity of pain now than they had experienced in the past. The reason for this is not clear, but Vervoort found that children tend to have higher pain expressions in the presence of caregivers than in the presence of strangers (who the researcher was). We should also consider reporting past pain experiences. As pointed out by Jaaniste, Noel, and von Baeyer [22] rating on “worst” pain is a complex socio-cognitive task and results in biases. Intrapersonal, interpersonal, and contextual factors can determine over-or under-estimations of previous pain scores.

Furthermore, a child’s perception of pain may be mediated by biological factors like sex and age, as well as socio-cultural factors—for example, differences in the responses of the environment (parents and peers) to reported pain [23]. In agreement with those on children with disease-related pain [24] or on the perception of painful procedures [25], and in contrast to studies on pain in childhood [26] and no statistically significant differences were found between the intensity of pain reported by girls and boys.

There were no significant differences in pain scores for parents, and children. Scores for worst and present pain can be compared with findings from other studies using the VAS for the assessment of chronic pain [11,18,21].

Pain coping strategies by children and adolescents seem to be intuitive and not based on professional support. Many studies have shown coping strategies change with age [27,28,29]. These changes can be explained based on Piaget’s theory of cognitive development. According to it, children aged 6–12 who participated in the study, are in the late preoperational stage (two to seven years), concrete stage (7–11 years), and at the beginning of the formal operational stage; the rest of the children participants (13–18 years) are formal operational children (11+ years old). We found differences in coping strategies in children and adolescents. Cognitive self-instruction strategies were found as the most often presented by younger children (6–12 years), as previously reported by Branson and Craig [30]. In contrary to other studies [31], where concrete operational children tend to use distraction, the need to involve parents when struggling with pain (Social support and action dimension), and attempts to solve the problem (for instance “ask for medicine”), were also indicated as strategies chosen in this age group. Adolescents (13–18 years) preferred both passive (resting) and active pain coping strategies: distraction and cognitive self-instruction. According to Herman [28], the last two strategies mentioned are more cognitively demanding but optimal for pain reduction. Adolescents prefer isolation during pain, which may reflect the need for independence from their parents, even when experiencing the pain [32]. Tendency to isolate while in pain in the adolescent group shown in the study corresponds with other findings: it has been shown that teenagers with tics are more likely to avoid communication with their parents because they feel responsible for family arguments as a result of their condition [33].

Children’s anger, irritability, or being cranky reported by parents are rather the effect of the pain, which corresponds with other studies dedicated to chronic pain [34].

Pain is a complex biopsychosocial phenomenon [26]. There is strong evidence that psychological treatments, principally relaxation and cognitive behavioral therapy, are highly effective in reducing the severity and frequency of chronic pain in children and adolescents [35]. Currently, relaxation therapy is a recommended component of behavioral treatments for tics [36]. We should consider that chronic pain can also induce depression [37]. Therefore, it is important that professionals working with people with TS also use pain relief techniques and teach children and their parents how to cope with pain.

In order to help people struggling with tic-related pain, researchers should also pay attention to brain mechanisms underlying Tourette and pain processes. The brain structures involved in tic generation (basal ganglia-cerebellar-thalamo-cortical system) [5], are also pointed out as recognized as playing a major role in the representation and modulation of pain experience [38]. That is why further research, for instance, using computational modeling of tic-related pain to design therapeutic interventions should be considered.

## 5. Conclusions

Pain should be recognized as a common comorbid aspect of tic disorders in childhood. There is a correlation between pain intensity and the severity of tics: pain may impair the everyday functioning of children with TS. Therapy must include the reduction of pain caused by tics—for this purpose it is necessary to develop and implement a holistic therapeutic program for children and adolescents with TS, which includes various aspects of health and well-being.

## Figures and Tables

**Table 1:** Classification of Pain in Relation to Tic Disorders.

Type of Pain	
Exertional	Muscular pain due to excessive contraction
	Skeletal or joint pain
	Neuropathic pain (due to spinal cord, radicular or peripheral nerve compression)
Traumatic	Pain in a body part struck by a moving limb
	Pain in a moving body part striking something nearby
	Self-mutilation (including biting)
	Pain from compulsive touching of hot or sharp objects
	Pain inflicted on others from tics or compulsions
Pain Caused by Suppression of Tics	
Pain Relieving Tics	

**Table 2:** The correlations between the scorings of pain intensity and tic severity.

		YGTSS
Child	Present pain	0.860 *
	Worst pain	0.744 *
Parent	Present pain	0.843 *
	Worst pain	0.772 *

* *p* < 0001.

**Table 3:** Present and worst pain on VAS (range 0–10) for children with TS and their parents.

	Child (*n* = 40) Median (Mean, *SD*)	Parent (*n* = 57) Median (Mean, *SD*)	*p*
Present pain	1.0 (2.0; 2.5)	1.0 (2.2; 2.6)	0.720
Worst pain	3.0 (3.1; 2.9)	4.0 (3.9; 3.3)	0.265

*p* < 0005.

**Table 4:** Present and worst pain on VAS (range 0–10) for boys and girls with TS.

	Boys (*n* = 31) Median (Mean, *SD*)	Girls (*n* = 9) Median (Mean, *SD*)	*p*
Present pain	1.0 (1.9; 2.3)	1.0 (2.5; 3.1)	0.726
Worst pain	3.0 (3.1; 2.9)	2.5 (3.2; 3.2)	0.824

*p* < 0.05.

**Table 5:** Present and worst pain on VAS (range 0–10) for mothers and fathers of children with TS.

	Mothers (*n* = 35) Median (Mean, *SD*)	Fathers (*n* = 22) Median (Mean, *SD*)	*p*
Present pain	1.0 (2.3; 2.8)	1.0 (1.9; 2.1)	0.940
Worst pain	4.0 (3.7; 3.3)	3.5 (3.9; 3.3)	0.561

*p* < 0.05.

**Table 6:** Pain descriptors (in percentages) by children with TS and their parents.

	Pain Descriptors (%)
Child	sharp (84)
	stinging (75)
	burning (63)
	pressure (54)
	exhausting (42)
	stabbing (29)
	tingling (17)
Parent	sharp (72)
	stinging (56)
	stabbing (54)
	pressure (39)
	nagging (21)

**Table 7:** Pain coping strategies in children with Tourette Syndrome (TS) and their parents.

		Child (5–12 years) (*n* = 9) (%)	Parent (*n* = 17) (%)
Pain Coping Strategies	Wish for it to go away	60.9	70.0
	Try not to think about the pain or ignore the pain	56.5	55.5
	Try to be brave and not say anything	56.5	63.0
	Tell my mother or father	56.5	62.9
	Play a game	52.2	37.0
	Have my mother, father or a friend sit with me	47.8	55.5
	Imagine I can make the pain or hurt disappear by myself	47.8	25.9
	Know that I can ask for something that will make the pain or hurt feel better	47.8	55.6
	Tell myself to be brave	43.4	55.5
	Tell myself that it will be alright	43.4	55.5
	Go to sleep until it feels better	43.4	59.2
	Go to bed	39.1	55.5
	Ask for medicine	39.1	33.3
	Ask for a hug or kiss	39.1	51.8
	Rub the sore spot	39.1	40.7

**Table 8:** Pain coping strategies in adolescents with Tourette Syndrome (TS) and their parents.

		Adolescent (13–18 years) (*n* = 15) (%)	Parent (*n* = 24) (%)
Pain Coping Strategies	Go to bed	76.5	76.7
	Ask to be left alone	76.5	46.7
	Lie down	70.6	70.0
	Sleep it off	70.6	77.7
	Try to be strong and not say anything	70.6	76.6
	Wish for it to go away	70.6	70.0
	Visit with my friends	64.7	53.3
	Play a game	64.7	63.4
	Try not to think about the pain or ignore the pain	64.7	70.0
	Tell myself that it will be alright	58.8	66.7
	Watch TV	58.8	70.0
	Rub the sore spot	58.8	50.0
	Imagine I can make the pain disappear by myself	52.9	46.6
	Cry or yell	35.5	40.0
	Get angry, irritable, or cranky	58.8	60.0
	Tell myself I can handle it	56.6	56.6
	Have my mother, father or a friend sit with me	47.1	63.3
	Breathe deeply	47.0	53.3
	Tell my parent(s)	52.7	66.7
	Know that I can ask for something that will make the pain feel better	29.4	60.0

## Data Availability

The data presented in this study are available on request from the corresponding author.
